# Effects of different roasting methods on formation of acrylamide in pistachio

**DOI:** 10.1002/fsn3.1588

**Published:** 2020-05-12

**Authors:** Sonia Asadi, Mehran Aalami, Shahram Shoeibi, Mehdi Kashaninejad, Mohammad Ghorbani, Mostafa Delavar

**Affiliations:** ^1^ Department of Food Science and Technology Gorgan University of Agricultural Sciences and Natural Resources Gorgan Iran; ^2^ Food and Drug Laboratories Research Center IRAN Food and Drug Administration (IFDA) Tehran Iran; ^3^ Department of Food Process Engineering Gorgan University of Agricultural Science and Natural Resources Gorgan Iran; ^4^ Department of Pharmacology Arak University of Medical Science Arak Iran

**Keywords:** acrylamide, infrared, microwave, pistachio, roasting

## Abstract

Drying and roasting are conventional processes in the nut industry. However, roasting as an important procedure in nuts manufacturing may cause some physicochemical changes in nuts. Acrylamide is one of these chemical compounds that is formed due to the roasting process. Acrylamide is known as a neurotoxicant, carcinogen, and reproductive toxicant. In this study, raw and salted pistachios were roasted under three conditions including hot‐air, infrared (IR), and microwave methods. Then, 80 pistachio kernels were analyzed by ultra‐high‐performance liquid chromatography. The results showed that all samples contained different ranges of acrylamide between 57 ± 0.86 and 851 ± 2.8 μg/kg. Besides, raw pistachios and sun‐dried pistachios also contained acrylamide, with the amount of 57 ± 0.86 and 93 ± 1.07 μg/kg, respectively. The highest acrylamide amount was found in raw pistachio (unsalted) roasted by IR method, while lower acrylamide amount observed in the microwave method. The amount of acrylamide in salted and roasted pistachios was less than just roasted pistachios under the same conditions. Finally, in all the treatments, increasing temperature, time, voltage, and power lead to an increase in acrylamide levels. The results showed that acrylamide in the roasted pistachios may cause health problems. This study presents a novel investigation in the effects of roasting conditions (temperature, power, voltage, and time) on acrylamide content in pistachios.

## Graphical abstract

In this study, raw and salted pistachios were roasted by three different methods including hot air, infrared, and microwave ovens. The amounts of acrylamide in salted and roasted pistachios were less than just roasted pistachios under the same conditions. In all treatments, increasing temperature, time, and power lead to an increase in acrylamide levels.

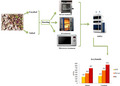

## 1 INTRODUCTION

Pistachio is the second most precious nonoil export product globally and the third largest export product of Iran. It is the most important agricultural product in Iran and the most expensive and delicious snack compared with the other ones and protein substances (Ardakani, 2006).

Acrylamide is produced during cooking or processing at high temperatures such as grilling, roasting, and frying (Süvari, Sivri, & Öksüz, 2017). International Agency of Research on Cancer in 2004 introduced acrylamide as a potential carcinogen substance for humans (Group 2A) and being neurotoxin and genotoxic (Capuano & Fogliano, 2011; Hogervorst et al., 2010; World Health Organization, 2002). The tolerable daily intake (TDI) of acrylamide is 40 μg/kg per day for neurotoxicity and 2.6 μg/kg per day for cancer. In addition to these toxicities, intake of acrylamide in the form of either dietary foods or foods from the environment is responsible for causing the Cardiac Developmental Toxicity (CDT) (Huang, Jiao, Wang, Xia, & Zhang, 2018).

In April 2002, the National Food Service of Sweden and the researchers of Stockholm University reported acrylamide compounds in foods. Acrylamide is a toxic and carcinogenic chemical compound, which is formed when the food is prepared in high temperature (>100°C) (Becalski, Lau, Lewis, & Seaman, 2003). Different mechanisms have been proposed for the formation of acrylamide due to the thermal process in which carbohydrates, proteins, lipids, amino acids, and possibly other compounds are associated with acrylamide formation (Eriksson, 2005).

Previous studies have shown that acrylamide is also formed in roasted nuts. Roasting is a conventional process in the nuts industry, which improves the aroma, texture, color, mouth feeling, and acceptance by the consumer. The enhancement in the product flavor depends on the roasting conditions, applied roasting technique, and type of nuts. “Roasting process” dehydrates and changes the food compound, which eventually increases the color, texture, and appearance of the nuts. The roasted nuts become rough, which is the characteristic of the roasted nuts. During the “roasting process,” the food lipid contents are oxidized and new compounds such as carbonyl are formed (Schlörmann et al., 2015). Enzyme and microorganism activities are inhibited by roasting (Özdemir et al., 2001). This is an advantage of the process since roasting also diminishes the Aflatoxin in pistachio (Yazdanpanah, Mohammadi, Abouhossain, & Cheraghali, 2005).

Pistachio is mostly salted and roasted in the food industry, but it is also applied as an ingredient or decoration in biscuits, bread, chocolate, ice cream, candy, fermented meat, sauces, puddings, and desserts (Ardakani, 2006; Hojjati, Noguera‐artiaga, Wojdy, & Carbonell‐barrachina, 2015). Several studies have been investigated the effect of “roasting” on physicochemical alternations in pistachio (Kahyaoglu, 2008; Nikzadeh & Sedaghat, 2008; Schlörmann et al., 2015). However, there is no reliable information regarding the effects of the roasting process on pistachio and acrylamide levels in roasted pistachios. In spite of the fact that acrylamide has a noxious effect on the nervous system, the risk level has not yet been determined (Arvanitoyannis & Dionisopoulou, 2014). Therefore, modification and controlling of acrylamide content would be more effective to improve human health and saving nutritional features. This indicates the importance of optimizing and correcting the process of roasting and evaluating acrylamide levels in roasted and salted pistachios, which is the main objective of this research.

There are three conventional methods regarding the roasting of nuts: hot air, microwave, and infrared (IR). In Iran, hot air is the typical roasting method. Further methods such as microwave, IR, and combined methods are also being used. In this research, the amount of acrylamide in roasted pistachios after using three roasting methods (hot air, infrared, and microwave) is measured.

## 2 MATERIALS AND METHODS

### 2.1 Chemical and reagents

Acrylamide (+99%) was purchased from Sigma. All solvents and reagents were of analytical grade and obtained from Merck. Ultrapure water was used for the experiments (Milli‐Q System, Millipore).

### 2.2 Standards

Acrylamide stock solution (100 mg/ml) was made in ultrapure (deionized) water. For additional standards, the stock solution was diluted using ultrapure water. All solutions were maintained at 4°C.

### 2.3 Pistachio samples

Raw pistachios, in coordination with the Iran Pistachio Research Institute, were purchased from a garden in Rafsanjan and kept at a cool place before further analysis. After peeling and sizing nuts using a caliper, pistachios with the equal dimensions were selected for testing.

### 2.4 Sample preparation

To prepare the samples, salted pistachios (weight ratio of 1 to 5) were soaked in water with 20% of NaCl and stirred slowly for 20 min. At that time, the samples were drained to remove the excess brine. Then, the brine surface was taken by a cloth filter. In the next step, the roasting was carried out with three methods on the raw and saline pistachios (Nikzadeh & Sedaghat, 2008).

### 2.5 Roasting conditions

In this study, the response surface method (RSM) in the form of a central composite design (CCD) was applied to predict the effect of roasting process (hot‐air, infrared, and microwave methods) on acrylamide formation in pistachios. The treatments were arranged in seven experiments based on the CCD and the central point repeated for five times (Kahyaoglu, 2008; Shakerardekani, Karim, Mohd Ghazali, & Chin, 2011).

The optimum condition was prepared based on sample type, laboratory situation, and the instruments. Independent variables that considered for hot‐air condition included temperature (100, 125, and 150°C) and the duration (5, 12.5, and 20 min). For infrared, it included the source voltage (75, 85, and 95 volts), irradiation duration (10, 20, and 30 min), and a constant 10 cm distance in the infrared (bidirectional source). For microwave, it included microwave power (180, 270, and 360 watts) and the time (12, 14, and 16 min). Before the roasting process, the devices were turned on to create a stable condition at the start of the process.

In treatment 1, the samples (batch size 250 g) were roasted in the laboratory oven (Memmert Gmbh and Co. KG). In treatments 2 and 3, they were roasted in infrared oven, which has two sources of radiation (built at Gorgan University), and microwave oven (Smary, China). Roasted pistachio samples were cooled down at room temperature. The samples were packaged in zip‐lock plastic bags and stored in a freezer until analysis.

### 2.6 Sample preparation procedure for acrylamide analysis

The sample preparation method for acrylamide analysis was performed according to the Süvari et al. (2017) method with some modifications (Omar, Elbashir, & Schmitz, 2015). First, pistachios were homogenized with a blender. Pistachios (5 g) were weighted in a 50‐ml centrifuged tube. 5 ml n‐hexane was added to the sample to remove the fat (defating), and then, samples were shaken by a vortex mixer for 5 min. Then, samples were treated with ultrapure water, acetonitrile (10 ml each), magnesium sulfate (5 g), and sodium chloride (1 g) mixture and immediately were shaken by a vortex mixer for 5 min. The suspension was centrifuged at 4,000 rpm for 10 min (Universal 320). In this stage, five separated layers were observed in the tube. The first layer of n‐hexane was removed. The second layer was acetonitrile, which contained acrylamide. This layer transferred into a 10‐ml falcon tube containing aluminum oxide (about 250 mg). After shaking for 1 min and centrifuging at 1,450 g for 5 min, all supernatant was transferred into the tube to evaporate under a moderate stream of nitrogen gas. At the end of the drying step, 500 μl of acetonitrile was added to the tube and dried again with nitrogen gas. Dried residues were diluted with 1,000 μl ultrapure water and then were shaken for 3 min and immersed into an ultrasonic bath for 2 min. Sample extract was filtered (0.20 μm) before being injected into a UHPLC autosampler cap vial.

### 2.7 Measurement of acrylamide

#### 2.7.1 Ultra‐high‐performance liquid chromatography–UV

The analytical procedure was set up by an ultra‐high‐performance liquid chromatography (KNAUER), which equipped with an automatic sampler (KNAUER) and coupled to a UV detector (KNAUER).

Separation was performed using a column Eurospher 100‐5 C18, 250 × 4.6 mm with precolumn. The injection was applied using an autosampler. The injection volume was 20 μl with a flow rate of 0.8 ml/min. A UV detector (210 nm) was applied in these experiments.

The characteristic of the syringe filter was 0.20 μm pore size and 25 mm diameter (MACHEREY‐NAGEL GmbH & Co. KG). The analytical column was Eurospher 100‐5 C18. The applied mobile phase consisted of ultrapure water and methanol with a ratio of 95:5 v/v. An optimum isocratic reversed‐phase separation has been used for detection. The mobile phase was degassed by sonication.

### 2.8 Method validation

#### 2.8.1 Selectivity

To calculate the selectivity of the extraction and HPLC methods, blank samples were extracted and analyzed according to the procedures mentioned above. The blank contains a specific amount of acrylamide (93 ± 1.07 μg/kg). In the first step, water was considered as a blank sample extracted by the extraction method and the obtained chromatogram was evaluated.

In this study, by considering the presence of acrylamide amount in the blank samples and also comparing the blank sample chromatograms with standard sample chromatogram, the selectivity of acrylamide detection procedure was calculated.

In addition, homogenized pistachios were spiked with 0.5, 2, and 5 μg/ml acrylamide and extracted in the same method by determination of the analytical recovery. The method's efficiency was determined by measuring the recovery of acrylamide. The final recovery was calculated according to the following equilibrium:

$$\text{Recovery}\;\%=\frac{\text{Experimental value}\;}{\text{Accepted value}}\times 100$$

#### 2.8.2 Linearity

Six different standard solutions of acrylamide in concentrations of 0.5, 1, 1.5, 2, 2.5, and 3 μg/ml were analyzed as triplicates. Average of the peak areas for each concentration was calculated.

#### 2.8.3 Limit of detection (LOD) and limit of quantitation (LOQ)

The lowest acrylamide concentration of acrylamide in pistachio that could be detected but not quantified accurately was defined as the limit of detection (LOD). The lowest acrylamide concentration that could be quantified by acceptable accuracy and precision was defined as the limit of quantitation (LOQ). The relation between signal and noise is applied to evaluate LOD and LOQ. The ratio of 3:1 was used for LOD, while the ratio of 10:1 was used for LOQ. The spiked acrylamide sample in the concentration of LOQ was injected to UHPLC for five times, and relative standard deviation of their peak areas and retention times of acrylamide were calculated.

### 2.9 Statistical analysis

The effect of roasting conditions on the acrylamide formation in pistachio nuts was performed by Microsoft Excel 2007.

A Minitab software version 15 determined confidence interval (CI) for data of this study.

The Design Expert Software version 11 was applied for running the RSM and optimum conditions. Data were analyzed for differences between means using SPSS version 16, and *p* < .05 is considered statistically significant.

## 3 RESULTS AND DISCUSSION

**FIGURE 1:**
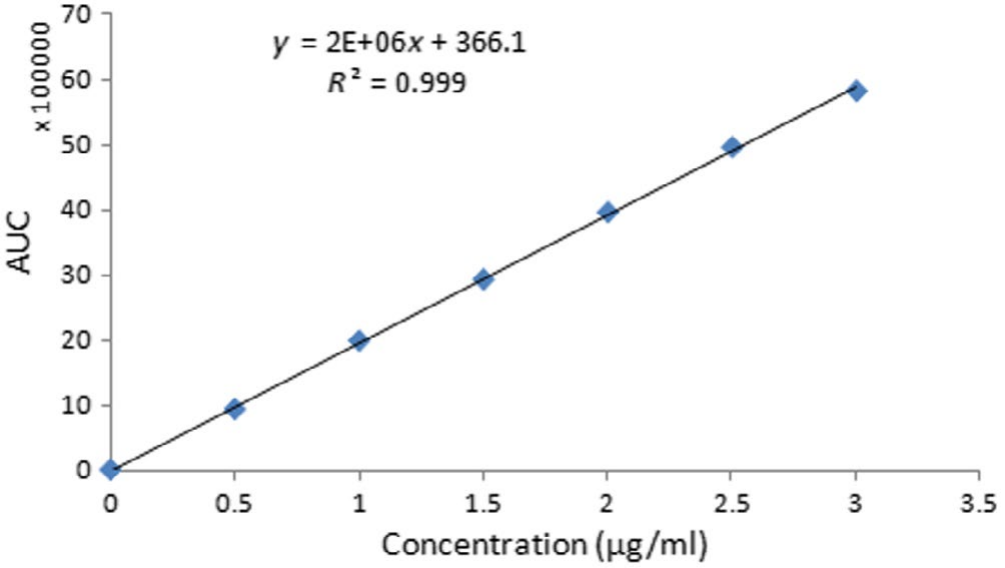
Calibration curve of acrylamide standards (0.5, 1, 1.5, 2, 2.5, and 3 μg/ml)

As described in Section 2.8.2, the calibration curve was plotted and linearity of the results was evaluated. The quantitation was established by using a calibration curve, and the formula is presented in Figure 1. The LOD, LOQ, and other evaluated parameters are presented in Table 1. Table 2 summarizes the results of recovery, percent error, standard deviation, and relative standard deviation of the method. These results showed that method validation is compatible for the determination of acrylamide in pistachio samples.

**TABLE 1:** The correlation coefficient, limit of detection, and limit of quantitation of the calibration curve

Method	*R* ^2^	LOD (μg/kg)	LOQ (μg/kg)
UHPLC/UV	.999	30	100

**TABLE 2:** The accepted value, experimental value, recovery, percent error, standard deviation, and relative standard deviation of the method

Method	Accepted value (μg/kg)	Experimental value (μg/kg)	Recovery %	E %	Std	RSD
UHPLC/UV	557	532	95.5	4.488	3.577	0.672

The acrylamide levels of roasted pistachios in the salted and unsalted types are shown in Table 3. Statistical data analysis indicated a significant relationship between the conditions of roasting and the acrylamide content.

**TABLE 3:** Acrylamide amount in salted and unsalted types of roasted pistachios (μg/kg)

Treatments	Samples	Minimum ± CI μg/kg	Maximum ± CI μg/kg
Hot air	Salted	130 ± 1.54	463 ± 1.63
	Unsalted	204 ± 2.12	594 ± 1.87
IR	Salted	242 ± 1.55	697 ± 2.31
	Unsalted	318 ± 1.91	851 ± 2.8
Microwave	Salted	105 ± 1.18	307 ± 1.62
	Unsalted	119 ± 1.66	344 ± 2.13

CI, Confidence interval.

Pistachios in salted and unsalted types with a different method of roasting contained different levels of acrylamide. Salted pistachios that roasted by microwave method at 180 w for 12 min contained the lowest acrylamide amounts (105 ± 1.18 μg/kg), whereas the IR‐roasted samples (95 V for 30 min) contained highest acrylamide amounts. In this survey, the level of acrylamide in roasted samples with IR was significantly higher than the other methods. Therefore, hot‐air and microwave methods in comparison with the first method contained a lower amount of acrylamide. Previous findings have shown that roasting with hot‐air method leads to acrylamide formation in different nuts.

A study by Zhang, Huang, Xiao, Seiber, and Mitchell (2011) and Lukac et al. (2007) showed that acrylamide was formed in almonds (Lukac et al., 2007; Zhang et al., 2011). Furthermore, Amrein et al. (2005) detected acrylamide on almond products (Amrein et al., 2005).

Except almonds, some research has been established on other nuts. For example, a study (2009) was conducted on roasted chest nut (Karasek, Wenzl, & Anklam, 2009).

Recently, a number of different nuts have been studied in 2015 and 2017, whereas Schlörmann et al. (2015) studied on hazelnuts, almonds, macadamia nuts, pistachios, and walnuts, among these roasted nuts, and acrylamide formation in almonds and pistachios was reported. The results in Süvari study are based on acrylamide formation in almonds, sunflower seeds, and peanuts (Süvari et al., 2017).

**FIGURE 2:**
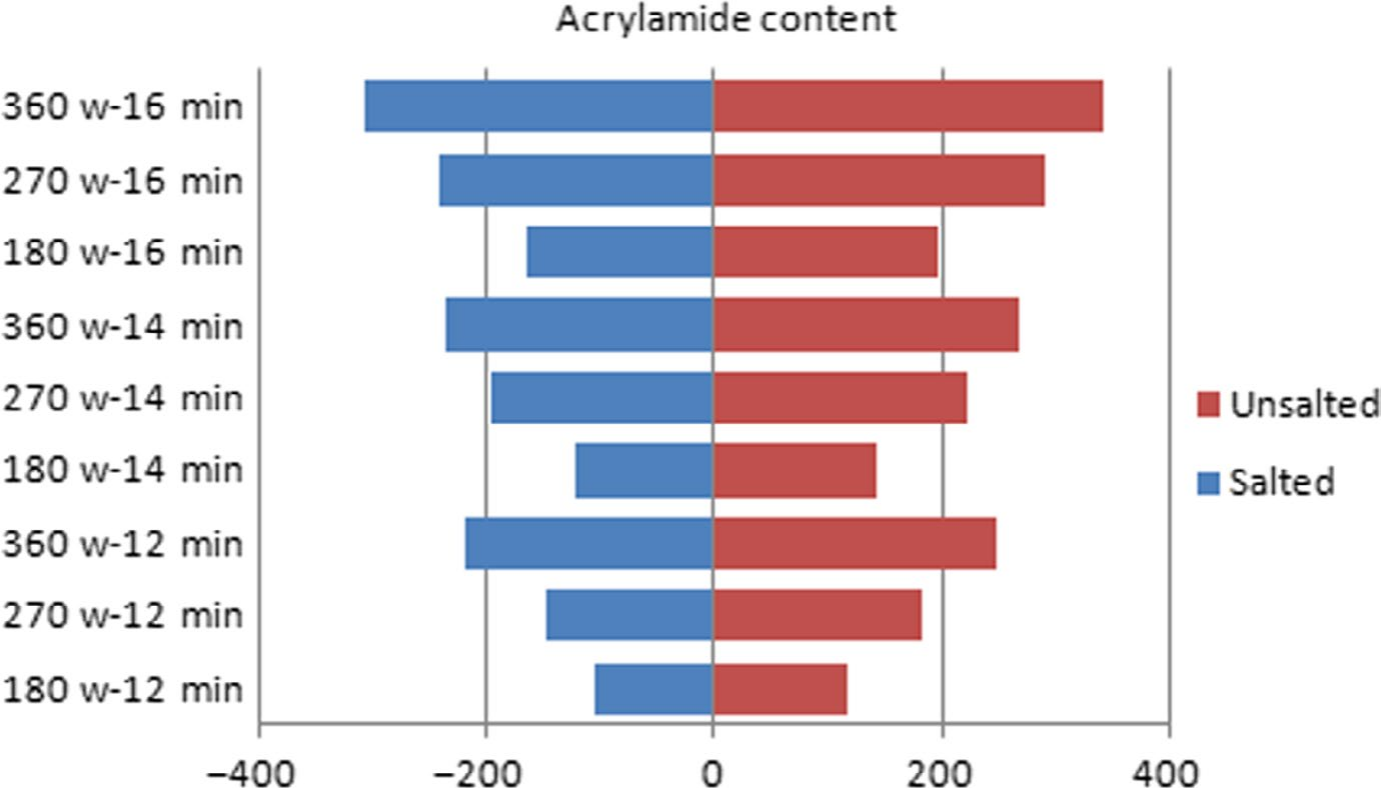
Effects of power and time in MW roasting on acrylamide formation amount in salted and unsalted pistachio types

**FIGURE 3:**
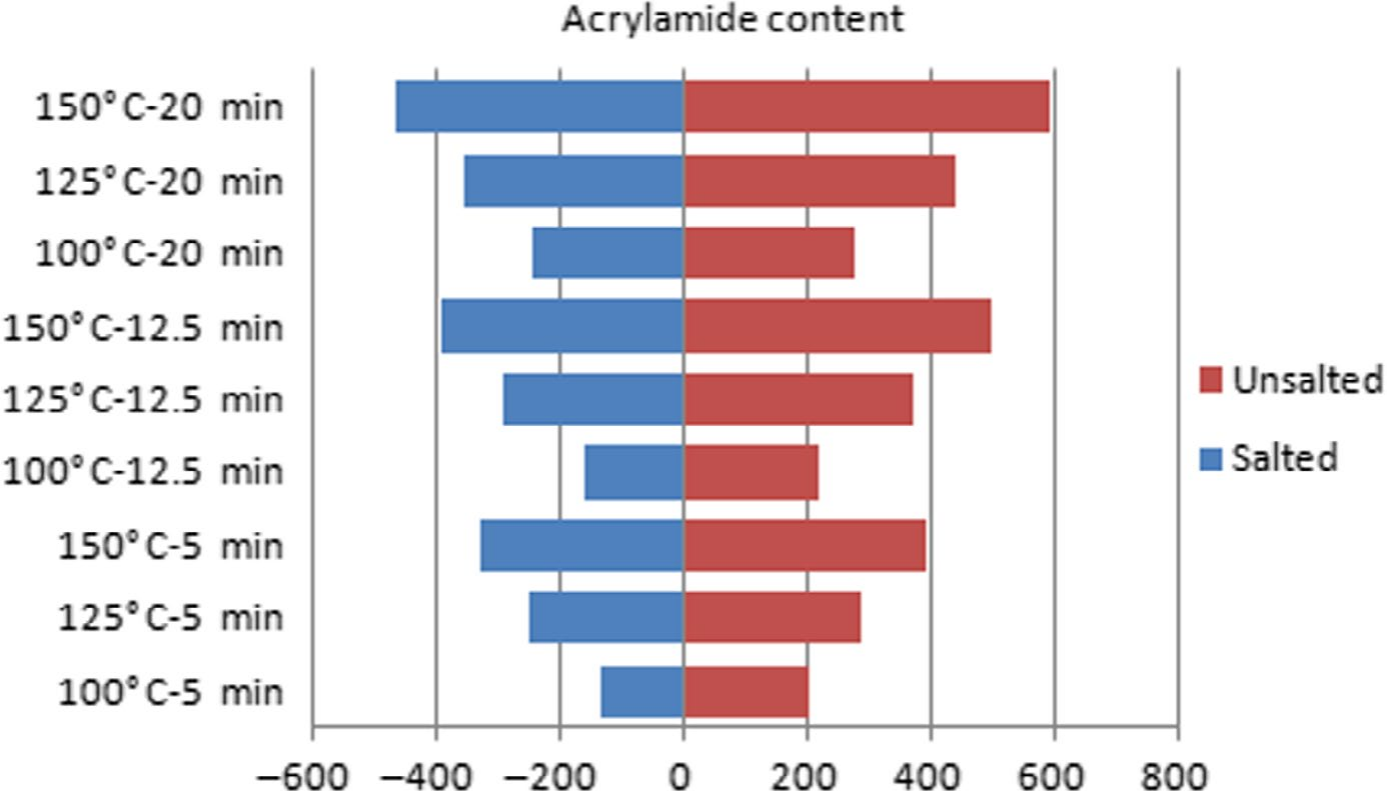
Effects of temperature and time in hot‐air roasting on acrylamide formation amount in salted and unsalted pistachio types

**FIGURE 4:**
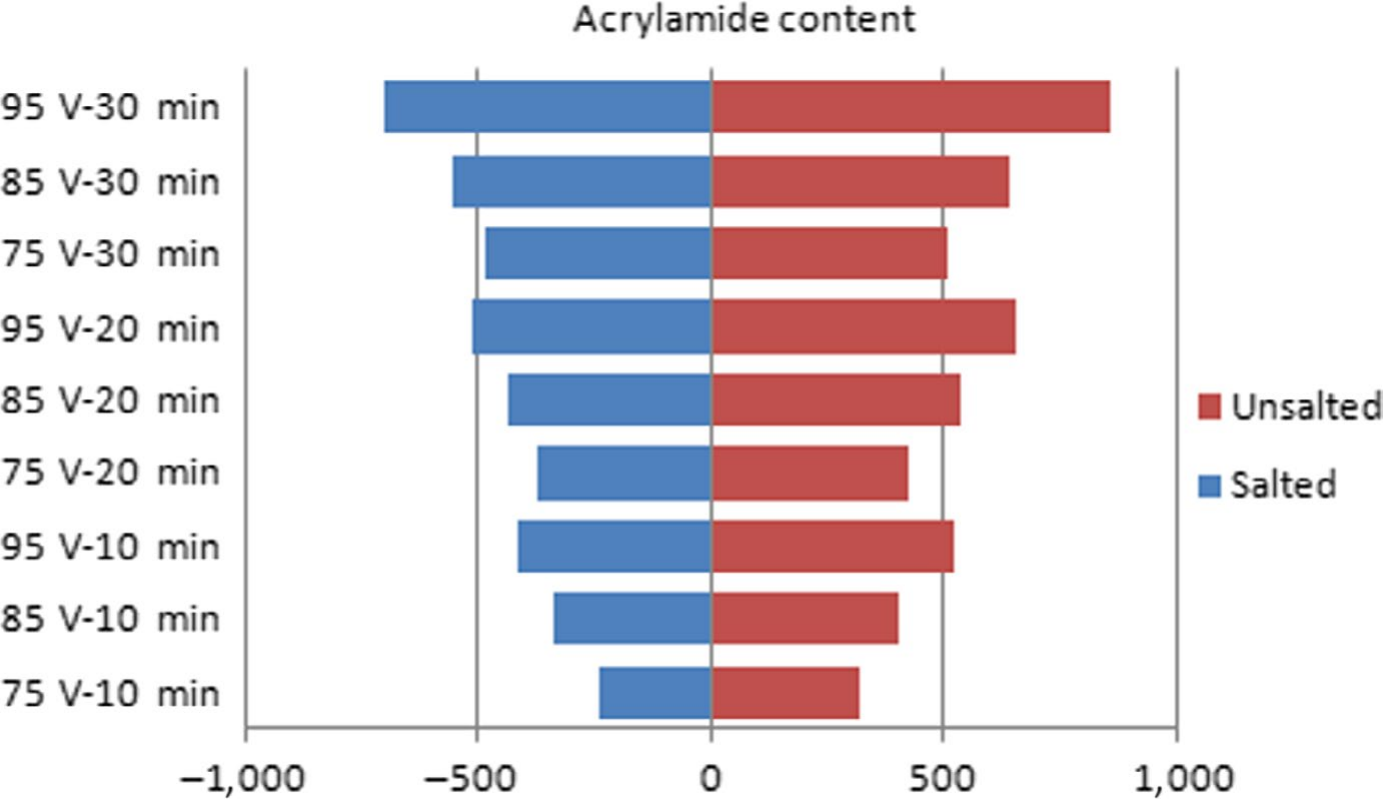
Effects of voltage and time in IR roasting on acrylamide formation amount in salted and unsalted pistachio types

However, there is no evidence of acrylamide formation in infrared and microwave methods. In order to investigate this relationship, roasting was done at different temperatures, powers, voltage, and time conditions. Figures 2, 3, 4 show the effect of temperature, power, voltage, and time on the formation of acrylamide in salted and unsalted pistachio kernels.

The amounts of acrylamide in salted/roasted pistachio were less than the unsalted/roasted pistachio. Lukac et al. (2007) also concluded that in the roasting conditions, the amount of acrylamide in roasted almonds that had higher moisture was less, because the existence of moisture during roasting decreased the temperature (Lukac et al., 2007).

According to the result, the temperature, power, voltage, and time of all roasting methods are directly related to the acrylamide content. Our result is inconsistent with the results reported by Schlörmann et al. (2015). In their study, the amount of acrylamide in roasted pistachios was reported as 14–88 μg/kg (140.8°C for 25 min to 185.1°C for 21 min) (Schlörmann et al., 2015). Özer, Kola, Altan, Duran, and Zorlugenç (2012) examined the amount of acrylamide in pistachios that is used in some Turkish traditional desserts. The acrylamide levels in two Baklava dessert samples were reported as 462 and 318 ng/g (Özer et al., 2012).

Based on the following results, the acrylamide level in the raw pistachios was 57 ± 0.86 μg/kg (<LOQ). In sun‐dried pistachios, acrylamide level also was increased up to 93 ± 1.07 μg/kg (<LOQ).

Nikzadeh and Sedaghat (2008) stated that roasting pistachio procedure has some benefits such as decreasing moisture content, fracture force, and hardness, which makes the pistachio crunchier. Shakerardekani et al. (2011) have demonstrated the positive effect of roasting pistachio such as the desired color, in addition to the mentioned features. It has been well demonstrated that “the roasting process” degrades Aflatoxins in contaminated pistachio (Yazdanpanah et al., 2005).

There are sufficient demonstrations about the benefits of roasting in reduction/elimination of dangerous microorganisms in nuts. However, the disadvantages of roasting methods are not well investigated. The present study showed that “roasting” could cause the acrylamide formation in pistachios. In other words, acrylamide formation enhanced along with increasing the temperature, power, voltage, and time of “roasting” method. The pistachios that roasted at 150°C for 20 min and also roasted in 95 volts for 30 min are known as the “over‐roasted” pistachios.

In this study, the lowest acrylamide amounts were generated in salted pistachios that were roasted by microwave (180 w for 12 min) and the highest acrylamide amounts were formed in the pistachio samples that were roasted by the IR method (95 volts for 30 min).

Generally, acrylamide is formed in both salted and roasted samples in all methods, but acrylamide content was less compared with the pistachios that were simply roasted. The reason for the lower acrylamide content in both salted and roasted pistachios is that they were somehow still wet (because the pistachios were soaked in water during preparation) so the part of the roasting time has been spent for drying the pistachios.

Roasting conditions significantly affected the amount of acrylamide formation (*p* < .05). By increasing the roasting grade, the amount of acrylamide in roasted pistachios was increased. Some consumers prefer “over‐roasted” nuts because of their taste and aroma. As mentioned above, the “over‐roasted” pistachios contain higher acrylamide level than less roasted ones; so, they may have carcinogenic effects on humans.

Since the acrylamide formation depends on the conditions of roasting, more research is required to find optimal roasting conditions to reduce the acrylamide amount and produce pistachios with low acrylamide content and desirable organoleptic characteristics.

## CONFLICT OF INTEREST

The authors declare that there is no conflict of interest regarding the publication of this article.

## ETHICAL APPROVAL

This article does not involve any human or animal testing.
